# Supplementary material for: Modeling glioblastoma heterogeneity as a dynamic network of cell states
Source: Mol Syst Biol. 2021 Sep 16;17(9):e10105. doi: 10.15252/msb.202010105 (PMC8444284; doi:10.15252/msb.202010105)
Supplement: Supplementary file 5 — Source Data for Figure 3 [file MSB-17-e10105-s001.zip › Figure3A_sourcedata/GSEA_3065/nosigdb_state3.GseaPreranked.1622718426028/RICHARDS_NOWAKOWSKI_SCIENCE_2017_RG_EARLY_UPREG.html]

Details for gene set RICHARDS\_NOWAKOWSKI\_SCIENCE\_2017\_RG\_EARLY\_UPREG[GSEA]

|  || Dataset | state3 |
| Phenotype | NoPhenotypeAvailable |
| Upregulated in class | na\_neg |
| GeneSet | RICHARDS\_NOWAKOWSKI\_SCIENCE\_2017\_RG\_EARLY\_UPREG |
| Enrichment Score (ES) | -0.4793803 |
| Normalized Enrichment Score (NES) | -2.0043645 |
| Nominal p-value | 0.0 |
| FDR q-value | 2.9847695E-4 |
| FWER p-Value | 0.003 |
Table: GSEA Results Summary

  

Fig 1: Enrichment plot: RICHARDS\_NOWAKOWSKI\_SCIENCE\_2017\_RG\_EARLY\_UPREG      
 Profile of the Running ES Score & Positions of GeneSet Members on the Rank Ordered List

  

| PROBE | GENE SYMBOL | GENE\_TITLE | RANK IN GENE LIST | RANK METRIC SCORE | RUNNING ES | CORE ENRICHMENT || 1 | CENPF |  |  | 4 | 0.619 | 0.0079 | No |
| 2 | HSP90B1 |  |  | 7 | 0.563 | 0.0152 | No |
| 3 | SYNE2 |  |  | 16 | 0.529 | 0.0214 | No |
| 4 | ASPM |  |  | 25 | 0.480 | 0.0269 | No |
| 5 | TOP2A |  |  | 42 | 0.448 | 0.0310 | No |
| 6 | MKI67 |  |  | 59 | 0.407 | 0.0345 | No |
| 7 | HSP90AA1 |  |  | 60 | 0.402 | 0.0400 | No |
| 8 | PON2 |  |  | 63 | 0.389 | 0.0450 | No |
| 9 | HSP90AB1 |  |  | 74 | 0.371 | 0.0488 | No |
| 10 | MDM4 |  |  | 92 | 0.351 | 0.0515 | No |
| 11 | FAT1 |  |  | 95 | 0.350 | 0.0559 | No |
| 12 | SEMA5A |  |  | 121 | 0.324 | 0.0573 | No |
| 13 | SMC2 |  |  | 125 | 0.322 | 0.0613 | No |
| 14 | CENPE |  |  | 126 | 0.321 | 0.0656 | No |
| 15 | TJP1 |  |  | 139 | 0.314 | 0.0684 | No |
| 16 | TTYH1 |  |  | 183 | 0.293 | 0.0671 | No |
| 17 | SPCS1 |  |  | 194 | 0.288 | 0.0698 | No |
| 18 | DLGAP5 |  |  | 207 | 0.279 | 0.0721 | No |
| 19 | SLC3A2 |  |  | 215 | 0.276 | 0.0750 | No |
| 20 | HELLS |  |  | 218 | 0.275 | 0.0785 | No |
| 21 | DNAJC1 |  |  | 227 | 0.272 | 0.0812 | No |
| 22 | SRPRB |  |  | 257 | 0.260 | 0.0812 | No |
| 23 | SMC4 |  |  | 265 | 0.255 | 0.0838 | No |
| 24 | ANLN |  |  | 281 | 0.249 | 0.0854 | No |
| 25 | ATAD2 |  |  | 282 | 0.249 | 0.0887 | No |
| 26 | JAM3 |  |  | 317 | 0.239 | 0.0879 | No |
| 27 | BRCA1 |  |  | 339 | 0.232 | 0.0885 | No |
| 28 | MCAM |  |  | 356 | 0.229 | 0.0896 | No |
| 29 | XPO1 |  |  | 390 | 0.220 | 0.0886 | No |
| 30 | PPP1R15B |  |  | 399 | 0.219 | 0.0906 | No |
| 31 | KIF20B |  |  | 411 | 0.216 | 0.0922 | No |
| 32 | KIF15 |  |  | 428 | 0.213 | 0.0931 | No |
| 33 | MAGED2 |  |  | 444 | 0.210 | 0.0942 | No |
| 34 | MIS18BP1 |  |  | 448 | 0.210 | 0.0966 | No |
| 35 | KIF11 |  |  | 451 | 0.209 | 0.0992 | No |
| 36 | ITGB8 |  |  | 463 | 0.206 | 0.1007 | No |
| 37 | FKBP10 |  |  | 481 | 0.201 | 0.1013 | No |
| 38 | MCM4 |  |  | 482 | 0.201 | 0.1040 | No |
| 39 | CREB5 |  |  | 497 | 0.199 | 0.1050 | No |
| 40 | SLC25A37 |  |  | 500 | 0.199 | 0.1075 | No |
| 41 | ECT2 |  |  | 527 | 0.195 | 0.1070 | No |
| 42 | CCNL1 |  |  | 529 | 0.194 | 0.1095 | No |
| 43 | TPX2 |  |  | 544 | 0.193 | 0.1104 | No |
| 44 | SREK1 |  |  | 578 | 0.189 | 0.1090 | No |
| 45 | NCAPG |  |  | 601 | 0.186 | 0.1088 | No |
| 46 | ABHD3 |  |  | 647 | 0.179 | 0.1058 | No |
| 47 | KIF23 |  |  | 662 | 0.177 | 0.1065 | No |
| 48 | ARHGAP11A |  |  | 702 | 0.170 | 0.1041 | No |
| 49 | AHI1 |  |  | 707 | 0.169 | 0.1059 | No |
| 50 | FGFR3 |  |  | 708 | 0.169 | 0.1082 | No |
| 51 | OFD1 |  |  | 714 | 0.168 | 0.1099 | No |
| 52 | TMEM97 |  |  | 740 | 0.165 | 0.1091 | No |
| 53 | NCAPG2 |  |  | 760 | 0.162 | 0.1090 | No |
| 54 | SLC5A3 |  |  | 812 | 0.156 | 0.1050 | No |
| 55 | FANCD2 |  |  | 813 | 0.156 | 0.1071 | No |
| 56 | PHLPP1 |  |  | 819 | 0.156 | 0.1086 | No |
| 57 | RBL1 |  |  | 844 | 0.153 | 0.1078 | No |
| 58 | MAD2L1 |  |  | 846 | 0.153 | 0.1097 | No |
| 59 | BUB1B |  |  | 857 | 0.152 | 0.1105 | No |
| 60 | KIF14 |  |  | 862 | 0.151 | 0.1121 | No |
| 61 | CNIH1 |  |  | 889 | 0.149 | 0.1110 | No |
| 62 | CDK1 |  |  | 915 | 0.147 | 0.1100 | No |
| 63 | HSPH1 |  |  | 937 | 0.144 | 0.1094 | No |
| 64 | HMGA2 |  |  | 948 | 0.143 | 0.1101 | No |
| 65 | MAP1B |  |  | 975 | 0.140 | 0.1089 | No |
| 66 | CEP135 |  |  | 1040 | 0.135 | 0.1030 | No |
| 67 | RBM39 |  |  | 1043 | 0.135 | 0.1046 | No |
| 68 | NDC80 |  |  | 1087 | 0.131 | 0.1012 | No |
| 69 | ATP1B1 |  |  | 1102 | 0.129 | 0.1012 | No |
| 70 | CCNB1 |  |  | 1152 | 0.126 | 0.0971 | No |
| 71 | TTK |  |  | 1177 | 0.123 | 0.0958 | No |
| 72 | RAD51AP1 |  |  | 1196 | 0.122 | 0.0953 | No |
| 73 | PSMD14 |  |  | 1227 | 0.120 | 0.0933 | No |
| 74 | LIFR |  |  | 1260 | 0.117 | 0.0911 | No |
| 75 | RTKN2 |  |  | 1285 | 0.116 | 0.0897 | No |
| 76 | SFRP1 |  |  | 1291 | 0.116 | 0.0907 | No |
| 77 | SAR1A |  |  | 1295 | 0.116 | 0.0919 | No |
| 78 | DCLRE1C |  |  | 1298 | 0.115 | 0.0932 | No |
| 79 | NUSAP1 |  |  | 1338 | 0.113 | 0.0900 | No |
| 80 | SPAG5 |  |  | 1357 | 0.111 | 0.0894 | No |
| 81 | CNTLN |  |  | 1378 | 0.110 | 0.0884 | No |
| 82 | TSPAN6 |  |  | 1430 | 0.107 | 0.0838 | No |
| 83 | PRR11 |  |  | 1465 | 0.105 | 0.0811 | No |
| 84 | IER5 |  |  | 1491 | 0.103 | 0.0795 | No |
| 85 | NUP155 |  |  | 1495 | 0.103 | 0.0805 | No |
| 86 | HES1 |  |  | 1501 | 0.103 | 0.0813 | No |
| 87 | GPC4 |  |  | 1512 | 0.102 | 0.0815 | No |
| 88 | DHX40 |  |  | 1521 | 0.102 | 0.0819 | No |
| 89 | PBK |  |  | 1524 | 0.102 | 0.0830 | No |
| 90 | ANP32E |  |  | 1548 | 0.101 | 0.0816 | No |
| 91 | SSB |  |  | 1559 | 0.100 | 0.0818 | No |
| 92 | PPIL4 |  |  | 1641 | 0.095 | 0.0733 | No |
| 93 | PEG3 |  |  | 1643 | 0.095 | 0.0745 | No |
| 94 | CREB3L2 |  |  | 1691 | 0.092 | 0.0701 | No |
| 95 | KIF18A |  |  | 1694 | 0.092 | 0.0711 | No |
| 96 | C1orf61 |  |  | 1703 | 0.091 | 0.0713 | No |
| 97 | VEGFA |  |  | 1723 | 0.090 | 0.0703 | No |
| 98 | MYADM |  |  | 1797 | 0.087 | 0.0627 | No |
| 99 | RNF139 |  |  | 1811 | 0.086 | 0.0623 | No |
| 100 | SCAF11 |  |  | 1822 | 0.085 | 0.0622 | No |
| 101 | TRA2A |  |  | 1849 | 0.084 | 0.0602 | No |
| 102 | AGO3 |  |  | 1854 | 0.084 | 0.0609 | No |
| 103 | MCM3 |  |  | 1918 | 0.080 | 0.0544 | No |
| 104 | INTS8 |  |  | 1973 | 0.077 | 0.0489 | No |
| 105 | KDM3A |  |  | 1980 | 0.077 | 0.0492 | No |
| 106 | KIF2C |  |  | 1985 | 0.077 | 0.0498 | No |
| 107 | ARHGEF10 |  |  | 2050 | 0.074 | 0.0431 | No |
| 108 | DPM1 |  |  | 2085 | 0.073 | 0.0400 | No |
| 109 | NOP58 |  |  | 2125 | 0.071 | 0.0363 | No |
| 110 | UHRF1 |  |  | 2136 | 0.071 | 0.0360 | No |
| 111 | CEP152 |  |  | 2138 | 0.071 | 0.0369 | No |
| 112 | HAUS8 |  |  | 2180 | 0.069 | 0.0329 | No |
| 113 | IGF2BP2 |  |  | 2210 | 0.068 | 0.0303 | No |
| 114 | CCDC18 |  |  | 2214 | 0.068 | 0.0308 | No |
| 115 | CDCA3 |  |  | 2232 | 0.067 | 0.0297 | No |
| 116 | ITGB3BP |  |  | 2263 | 0.066 | 0.0270 | No |
| 117 | ADAMTS9 |  |  | 2279 | 0.065 | 0.0261 | No |
| 118 | CHORDC1 |  |  | 2288 | 0.065 | 0.0260 | No |
| 119 | ID4 |  |  | 2306 | 0.064 | 0.0248 | No |
| 120 | HSPA4L |  |  | 2311 | 0.064 | 0.0252 | No |
| 121 | THUMPD1 |  |  | 2352 | 0.063 | 0.0212 | No |
| 122 | PTBP1 |  |  | 2359 | 0.062 | 0.0213 | No |
| 123 | ADNP |  |  | 2362 | 0.062 | 0.0219 | No |
| 124 | NOTCH3 |  |  | 2367 | 0.062 | 0.0223 | No |
| 125 | DYRK1A |  |  | 2413 | 0.060 | 0.0177 | No |
| 126 | NFYB |  |  | 2471 | 0.058 | 0.0116 | No |
| 127 | CHEK1 |  |  | 2475 | 0.058 | 0.0120 | No |
| 128 | BARD1 |  |  | 2514 | 0.056 | 0.0082 | No |
| 129 | CCNA2 |  |  | 2529 | 0.056 | 0.0073 | No |
| 130 | NPM1 |  |  | 2533 | 0.056 | 0.0077 | No |
| 131 | KIF22 |  |  | 2545 | 0.055 | 0.0071 | No |
| 132 | UBE2T |  |  | 2599 | 0.054 | 0.0015 | No |
| 133 | BTG3 |  |  | 2610 | 0.053 | 0.0010 | No |
| 134 | GPBP1L1 |  |  | 2628 | 0.052 | -0.0003 | No |
| 135 | WEE1 |  |  | 2687 | 0.050 | -0.0066 | No |
| 136 | HSPD1 |  |  | 2700 | 0.050 | -0.0074 | No |
| 137 | RDH10 |  |  | 2702 | 0.050 | -0.0068 | No |
| 138 | MDM1 |  |  | 2738 | 0.049 | -0.0104 | No |
| 139 | TNFRSF19 |  |  | 2761 | 0.048 | -0.0124 | No |
| 140 | ZEB1 |  |  | 2764 | 0.048 | -0.0120 | No |
| 141 | RACGAP1 |  |  | 2769 | 0.048 | -0.0118 | No |
| 142 | DUSP16 |  |  | 2824 | 0.046 | -0.0177 | No |
| 143 | TAF8 |  |  | 2833 | 0.046 | -0.0180 | No |
| 144 | RRM2 |  |  | 2835 | 0.046 | -0.0175 | No |
| 145 | FXR1 |  |  | 2873 | 0.045 | -0.0213 | No |
| 146 | MIER1 |  |  | 2919 | 0.043 | -0.0262 | No |
| 147 | KNSTRN |  |  | 2976 | 0.042 | -0.0323 | No |
| 148 | EFNB1 |  |  | 2999 | 0.041 | -0.0344 | No |
| 149 | LDHB |  |  | 3031 | 0.040 | -0.0376 | No |
| 150 | GPCPD1 |  |  | 3151 | 0.037 | -0.0514 | No |
| 151 | NUP50 |  |  | 3160 | 0.037 | -0.0519 | No |
| 152 | KIF4A |  |  | 3195 | 0.036 | -0.0555 | No |
| 153 | CKAP2 |  |  | 3236 | 0.035 | -0.0598 | No |
| 154 | SRSF3 |  |  | 3283 | 0.034 | -0.0649 | No |
| 155 | TLK1 |  |  | 3326 | 0.032 | -0.0695 | No |
| 156 | CBFB |  |  | 3344 | 0.032 | -0.0711 | No |
| 157 | ZNF134 |  |  | 3373 | 0.031 | -0.0740 | No |
| 158 | SPC24 |  |  | 3409 | 0.030 | -0.0778 | No |
| 159 | ZFHX4 |  |  | 3437 | 0.029 | -0.0807 | No |
| 160 | HSPA1B |  |  | 3440 | 0.029 | -0.0805 | No |
| 161 | TIMELESS |  |  | 3497 | 0.028 | -0.0869 | No |
| 162 | CCNB2 |  |  | 3502 | 0.028 | -0.0870 | No |
| 163 | KIAA1217 |  |  | 3533 | 0.027 | -0.0902 | No |
| 164 | RPF2 |  |  | 3573 | 0.026 | -0.0945 | No |
| 165 | SPC25 |  |  | 3575 | 0.026 | -0.0943 | No |
| 166 | TMEM170A |  |  | 3589 | 0.026 | -0.0955 | No |
| 167 | GSK3B |  |  | 3606 | 0.025 | -0.0971 | No |
| 168 | TOPORS |  |  | 3676 | 0.023 | -0.1051 | No |
| 169 | CKAP2L |  |  | 3787 | 0.020 | -0.1180 | No |
| 170 | SALL1 |  |  | 3800 | 0.020 | -0.1192 | No |
| 171 | CCT4 |  |  | 3867 | 0.018 | -0.1268 | No |
| 172 | ZNF516 |  |  | 3872 | 0.018 | -0.1271 | No |
| 173 | PRC1 |  |  | 3878 | 0.018 | -0.1274 | No |
| 174 | PTAR1 |  |  | 3879 | 0.018 | -0.1272 | No |
| 175 | PIK3R3 |  |  | 3885 | 0.018 | -0.1276 | No |
| 176 | SNRPA1 |  |  | 3957 | 0.016 | -0.1359 | No |
| 177 | PCNA |  |  | 3986 | 0.016 | -0.1390 | No |
| 178 | PDCD4 |  |  | 4013 | 0.015 | -0.1419 | No |
| 179 | MRPL47 |  |  | 4072 | 0.013 | -0.1487 | No |
| 180 | KIF5B |  |  | 4156 | 0.011 | -0.1585 | No |
| 181 | FOXN2 |  |  | 4204 | 0.009 | -0.1641 | No |
| 182 | ASNS |  |  | 4228 | 0.009 | -0.1667 | No |
| 183 | REST |  |  | 4235 | 0.009 | -0.1673 | No |
| 184 | PSIP1 |  |  | 4250 | 0.008 | -0.1689 | No |
| 185 | PPA1 |  |  | 4255 | 0.008 | -0.1693 | No |
| 186 | CENPK |  |  | 4433 | 0.003 | -0.1905 | No |
| 187 | HMGB3 |  |  | 4445 | 0.003 | -0.1918 | No |
| 188 | PBDC1 |  |  | 4469 | 0.002 | -0.1945 | No |
| 189 | ZNF844 |  |  | 4476 | 0.002 | -0.1952 | No |
| 190 | TCF3 |  |  | 4499 | 0.001 | -0.1978 | No |
| 191 | SOX9 |  |  | 4503 | 0.001 | -0.1982 | No |
| 192 | TMEM258 |  |  | 4509 | 0.001 | -0.1987 | No |
| 193 | MSI1 |  |  | 4661 | -0.003 | -0.2168 | No |
| 194 | RFX4 |  |  | 4672 | -0.003 | -0.2180 | No |
| 195 | DDX39A |  |  | 4706 | -0.004 | -0.2219 | No |
| 196 | TUBE1 |  |  | 4733 | -0.004 | -0.2250 | No |
| 197 | EAF1 |  |  | 4767 | -0.005 | -0.2289 | No |
| 198 | G3BP1 |  |  | 4777 | -0.005 | -0.2299 | No |
| 199 | GLI3 |  |  | 4782 | -0.006 | -0.2303 | No |
| 200 | ZDHHC2 |  |  | 4789 | -0.006 | -0.2309 | No |
| 201 | OSER1 |  |  | 4825 | -0.007 | -0.2350 | No |
| 202 | ZNF217 |  |  | 4854 | -0.007 | -0.2383 | No |
| 203 | DBF4 |  |  | 4926 | -0.009 | -0.2467 | No |
| 204 | IRF2BP2 |  |  | 4927 | -0.009 | -0.2466 | No |
| 205 | LEF1 |  |  | 4928 | -0.009 | -0.2465 | No |
| 206 | ZWINT |  |  | 4930 | -0.009 | -0.2465 | No |
| 207 | EZH2 |  |  | 4952 | -0.010 | -0.2489 | No |
| 208 | PPP1R15A |  |  | 4983 | -0.010 | -0.2523 | No |
| 209 | YAP1 |  |  | 5041 | -0.012 | -0.2590 | No |
| 210 | RC3H2 |  |  | 5101 | -0.014 | -0.2659 | No |
| 211 | MTHFD2L |  |  | 5124 | -0.014 | -0.2684 | No |
| 212 | WDR43 |  |  | 5243 | -0.017 | -0.2823 | No |
| 213 | WDR36 |  |  | 5248 | -0.017 | -0.2826 | No |
| 214 | SP3 |  |  | 5332 | -0.019 | -0.2923 | No |
| 215 | WBP4 |  |  | 5359 | -0.020 | -0.2951 | No |
| 216 | TCF7L1 |  |  | 5378 | -0.020 | -0.2970 | No |
| 217 | HMGB2 |  |  | 5479 | -0.023 | -0.3087 | No |
| 218 | PSMA3 |  |  | 5498 | -0.024 | -0.3105 | No |
| 219 | EIF3J |  |  | 5590 | -0.027 | -0.3211 | No |
| 220 | DARS |  |  | 5647 | -0.028 | -0.3275 | No |
| 221 | PBX3 |  |  | 5653 | -0.029 | -0.3277 | No |
| 222 | TBL1X |  |  | 5658 | -0.029 | -0.3278 | No |
| 223 | TPM1 |  |  | 5672 | -0.029 | -0.3289 | No |
| 224 | EIF1AD |  |  | 5682 | -0.029 | -0.3296 | No |
| 225 | NFKBIA |  |  | 5684 | -0.029 | -0.3293 | No |
| 226 | TP53 |  |  | 5700 | -0.030 | -0.3307 | No |
| 227 | HDAC9 |  |  | 5716 | -0.030 | -0.3321 | No |
| 228 | RAP2C |  |  | 5757 | -0.032 | -0.3365 | No |
| 229 | MED30 |  |  | 5767 | -0.032 | -0.3372 | No |
| 230 | NEK4 |  |  | 5772 | -0.032 | -0.3372 | No |
| 231 | IMMP1L |  |  | 5866 | -0.035 | -0.3479 | No |
| 232 | MND1 |  |  | 5880 | -0.035 | -0.3490 | No |
| 233 | TYW3 |  |  | 5888 | -0.035 | -0.3494 | No |
| 234 | ZCCHC3 |  |  | 5890 | -0.035 | -0.3490 | No |
| 235 | TCF7L2 |  |  | 5935 | -0.037 | -0.3538 | No |
| 236 | USP1 |  |  | 5963 | -0.038 | -0.3565 | No |
| 237 | CDC42EP4 |  |  | 6004 | -0.040 | -0.3608 | No |
| 238 | RSL24D1 |  |  | 6017 | -0.040 | -0.3617 | No |
| 239 | IFT74 |  |  | 6066 | -0.042 | -0.3669 | No |
| 240 | RSRC2 |  |  | 6096 | -0.043 | -0.3698 | No |
| 241 | TWISTNB |  |  | 6141 | -0.044 | -0.3745 | No |
| 242 | ATF3 |  |  | 6159 | -0.045 | -0.3759 | No |
| 243 | AHSA1 |  |  | 6179 | -0.046 | -0.3776 | No |
| 244 | PPM1D |  |  | 6214 | -0.047 | -0.3811 | No |
| 245 | MDK |  |  | 6242 | -0.048 | -0.3837 | No |
| 246 | GMNN |  |  | 6392 | -0.052 | -0.4008 | No |
| 247 | HIST1H1D |  |  | 6428 | -0.054 | -0.4043 | No |
| 248 | TFAP2C |  |  | 6436 | -0.054 | -0.4044 | No |
| 249 | PSMA1 |  |  | 6529 | -0.057 | -0.4147 | No |
| 250 | CHMP1B |  |  | 6554 | -0.059 | -0.4168 | No |
| 251 | CKS2 |  |  | 6569 | -0.059 | -0.4177 | No |
| 252 | FAM162A |  |  | 6572 | -0.060 | -0.4171 | No |
| 253 | TAF7 |  |  | 6581 | -0.060 | -0.4173 | No |
| 254 | PSMB7 |  |  | 6586 | -0.060 | -0.4170 | No |
| 255 | FDFT1 |  |  | 6637 | -0.063 | -0.4221 | No |
| 256 | ERRFI1 |  |  | 6678 | -0.065 | -0.4260 | No |
| 257 | TMSB15A |  |  | 6711 | -0.067 | -0.4290 | No |
| 258 | MRPL50 |  |  | 6739 | -0.068 | -0.4313 | No |
| 259 | CENPW |  |  | 6750 | -0.068 | -0.4316 | No |
| 260 | ZFP36L2 |  |  | 6774 | -0.070 | -0.4334 | No |
| 261 | ATF4 |  |  | 6789 | -0.070 | -0.4342 | No |
| 262 | ETF1 |  |  | 6805 | -0.071 | -0.4350 | No |
| 263 | RNPS1 |  |  | 6814 | -0.072 | -0.4350 | No |
| 264 | RPL22L1 |  |  | 6882 | -0.075 | -0.4420 | No |
| 265 | TGIF1 |  |  | 6888 | -0.075 | -0.4416 | No |
| 266 | ZFP36L1 |  |  | 6893 | -0.076 | -0.4411 | No |
| 267 | DDIT3 |  |  | 6918 | -0.077 | -0.4429 | No |
| 268 | HEY1 |  |  | 6933 | -0.077 | -0.4436 | No |
| 269 | CASP3 |  |  | 6969 | -0.079 | -0.4467 | No |
| 270 | USP39 |  |  | 6997 | -0.080 | -0.4489 | No |
| 271 | COPS8 |  |  | 7011 | -0.082 | -0.4493 | No |
| 272 | TIPARP |  |  | 7051 | -0.084 | -0.4529 | No |
| 273 | KCTD6 |  |  | 7072 | -0.085 | -0.4541 | No |
| 274 | GPN1 |  |  | 7075 | -0.085 | -0.4532 | No |
| 275 | HIST1H1A |  |  | 7091 | -0.086 | -0.4539 | No |
| 276 | UBB |  |  | 7097 | -0.087 | -0.4533 | No |
| 277 | ATP6V0E1 |  |  | 7106 | -0.088 | -0.4531 | No |
| 278 | RBBP8 |  |  | 7128 | -0.089 | -0.4544 | No |
| 279 | RYBP |  |  | 7132 | -0.089 | -0.4536 | No |
| 280 | TCP1 |  |  | 7137 | -0.090 | -0.4528 | No |
| 281 | CAPZA1 |  |  | 7157 | -0.091 | -0.4539 | No |
| 282 | BOC |  |  | 7182 | -0.092 | -0.4555 | No |
| 283 | CCDC59 |  |  | 7197 | -0.093 | -0.4560 | No |
| 284 | BTBD1 |  |  | 7223 | -0.095 | -0.4577 | No |
| 285 | HMGB1 |  |  | 7231 | -0.096 | -0.4572 | No |
| 286 | PIN4 |  |  | 7252 | -0.097 | -0.4583 | No |
| 287 | ENO1 |  |  | 7257 | -0.097 | -0.4575 | No |
| 288 | PPP4R2 |  |  | 7306 | -0.101 | -0.4619 | No |
| 289 | DPY30 |  |  | 7347 | -0.104 | -0.4653 | No |
| 290 | ARRDC3 |  |  | 7386 | -0.107 | -0.4684 | No |
| 291 | PAPOLA |  |  | 7410 | -0.109 | -0.4697 | No |
| 292 | EIF1B |  |  | 7425 | -0.110 | -0.4699 | No |
| 293 | BET1 |  |  | 7433 | -0.111 | -0.4693 | No |
| 294 | SNRPD1 |  |  | 7463 | -0.113 | -0.4712 | No |
| 295 | RBM8A |  |  | 7532 | -0.120 | -0.4778 | Yes |
| 296 | NAP1L1 |  |  | 7538 | -0.121 | -0.4767 | Yes |
| 297 | SLC25A3 |  |  | 7543 | -0.122 | -0.4756 | Yes |
| 298 | SVIP |  |  | 7544 | -0.122 | -0.4739 | Yes |
| 299 | ACAA2 |  |  | 7550 | -0.122 | -0.4729 | Yes |
| 300 | CKS1B |  |  | 7569 | -0.124 | -0.4734 | Yes |
| 301 | ZFAND2A |  |  | 7576 | -0.125 | -0.4724 | Yes |
| 302 | PSMB3 |  |  | 7602 | -0.128 | -0.4737 | Yes |
| 303 | NOL7 |  |  | 7603 | -0.128 | -0.4720 | Yes |
| 304 | DUT |  |  | 7608 | -0.128 | -0.4707 | Yes |
| 305 | YWHAE |  |  | 7628 | -0.131 | -0.4712 | Yes |
| 306 | SLIRP |  |  | 7646 | -0.132 | -0.4715 | Yes |
| 307 | CKB |  |  | 7659 | -0.134 | -0.4711 | Yes |
| 308 | SNRPB |  |  | 7671 | -0.136 | -0.4706 | Yes |
| 309 | CACYBP |  |  | 7708 | -0.141 | -0.4731 | Yes |
| 310 | PUF60 |  |  | 7729 | -0.143 | -0.4735 | Yes |
| 311 | BIRC5 |  |  | 7735 | -0.144 | -0.4722 | Yes |
| 312 | CENPN |  |  | 7756 | -0.147 | -0.4726 | Yes |
| 313 | HIST1H1E |  |  | 7780 | -0.149 | -0.4734 | Yes |
| 314 | CENPU |  |  | 7781 | -0.149 | -0.4714 | Yes |
| 315 | TYMS |  |  | 7792 | -0.150 | -0.4706 | Yes |
| 316 | EEF1D |  |  | 7817 | -0.154 | -0.4714 | Yes |
| 317 | CMBL |  |  | 7837 | -0.156 | -0.4715 | Yes |
| 318 | CCDC25 |  |  | 7842 | -0.157 | -0.4699 | Yes |
| 319 | GLO1 |  |  | 7844 | -0.157 | -0.4679 | Yes |
| 320 | TOMM5 |  |  | 7849 | -0.158 | -0.4663 | Yes |
| 321 | CTNNAL1 |  |  | 7886 | -0.164 | -0.4684 | Yes |
| 322 | HIST1H1B |  |  | 7908 | -0.168 | -0.4686 | Yes |
| 323 | SCAND1 |  |  | 7912 | -0.169 | -0.4667 | Yes |
| 324 | HIST1H1C |  |  | 7917 | -0.170 | -0.4649 | Yes |
| 325 | HSPE1 |  |  | 7918 | -0.170 | -0.4626 | Yes |
| 326 | H2AFZ |  |  | 7924 | -0.171 | -0.4609 | Yes |
| 327 | RAN |  |  | 7979 | -0.181 | -0.4650 | Yes |
| 328 | SNRPE |  |  | 7980 | -0.181 | -0.4625 | Yes |
| 329 | PPP1CC |  |  | 7981 | -0.182 | -0.4601 | Yes |
| 330 | CDKN3 |  |  | 7998 | -0.184 | -0.4595 | Yes |
| 331 | WTAP |  |  | 8000 | -0.186 | -0.4571 | Yes |
| 332 | HIST1H4C |  |  | 8075 | -0.198 | -0.4633 | Yes |
| 333 | PGAM1 |  |  | 8093 | -0.203 | -0.4626 | Yes |
| 334 | PNRC1 |  |  | 8133 | -0.212 | -0.4645 | Yes |
| 335 | TAF9 |  |  | 8142 | -0.214 | -0.4626 | Yes |
| 336 | BNIP3 |  |  | 8151 | -0.215 | -0.4606 | Yes |
| 337 | RHEB |  |  | 8224 | -0.234 | -0.4661 | Yes |
| 338 | CNN3 |  |  | 8228 | -0.234 | -0.4633 | Yes |
| 339 | NDUFB1 |  |  | 8241 | -0.238 | -0.4616 | Yes |
| 340 | GSTP1 |  |  | 8296 | -0.257 | -0.4646 | Yes |
| 341 | RPL15 |  |  | 8308 | -0.260 | -0.4624 | Yes |
| 342 | UQCRFS1 |  |  | 8310 | -0.260 | -0.4590 | Yes |
| 343 | TRIAP1 |  |  | 8318 | -0.262 | -0.4563 | Yes |
| 344 | PEA15 |  |  | 8347 | -0.272 | -0.4560 | Yes |
| 345 | RPL5 |  |  | 8352 | -0.275 | -0.4528 | Yes |
| 346 | PTTG1 |  |  | 8366 | -0.279 | -0.4506 | Yes |
| 347 | DEK |  |  | 8376 | -0.284 | -0.4479 | Yes |
| 348 | SRP14 |  |  | 8397 | -0.293 | -0.4463 | Yes |
| 349 | CYCS |  |  | 8405 | -0.297 | -0.4432 | Yes |
| 350 | NDUFA6 |  |  | 8407 | -0.298 | -0.4393 | Yes |
| 351 | PRDX6 |  |  | 8410 | -0.300 | -0.4355 | Yes |
| 352 | PKM |  |  | 8420 | -0.301 | -0.4325 | Yes |
| 353 | RPL18 |  |  | 8424 | -0.304 | -0.4288 | Yes |
| 354 | H1FX |  |  | 8441 | -0.311 | -0.4265 | Yes |
| 355 | SNRPG |  |  | 8443 | -0.312 | -0.4224 | Yes |
| 356 | HMGN3 |  |  | 8452 | -0.316 | -0.4191 | Yes |
| 357 | RPL14 |  |  | 8458 | -0.320 | -0.4154 | Yes |
| 358 | RPL13A |  |  | 8472 | -0.325 | -0.4126 | Yes |
| 359 | ANAPC16 |  |  | 8480 | -0.331 | -0.4090 | Yes |
| 360 | CSNK2B |  |  | 8485 | -0.332 | -0.4050 | Yes |
| 361 | RPS7 |  |  | 8486 | -0.333 | -0.4005 | Yes |
| 362 | RPL24 |  |  | 8492 | -0.336 | -0.3966 | Yes |
| 363 | MYL6 |  |  | 8495 | -0.339 | -0.3923 | Yes |
| 364 | RPL7 |  |  | 8505 | -0.342 | -0.3887 | Yes |
| 365 | RPL10A |  |  | 8509 | -0.344 | -0.3845 | Yes |
| 366 | SIVA1 |  |  | 8511 | -0.344 | -0.3799 | Yes |
| 367 | HMGA1 |  |  | 8519 | -0.346 | -0.3761 | Yes |
| 368 | RPS24 |  |  | 8521 | -0.347 | -0.3716 | Yes |
| 369 | RPS27L |  |  | 8528 | -0.351 | -0.3676 | Yes |
| 370 | NACA |  |  | 8534 | -0.354 | -0.3634 | Yes |
| 371 | RPL23 |  |  | 8539 | -0.357 | -0.3591 | Yes |
| 372 | RPS21 |  |  | 8544 | -0.361 | -0.3547 | Yes |
| 373 | YBX3 |  |  | 8545 | -0.362 | -0.3498 | Yes |
| 374 | HSPB1 |  |  | 8572 | -0.383 | -0.3478 | Yes |
| 375 | GNG5 |  |  | 8575 | -0.384 | -0.3428 | Yes |
| 376 | ODC1 |  |  | 8576 | -0.385 | -0.3377 | Yes |
| 377 | RPL21 |  |  | 8588 | -0.393 | -0.3337 | Yes |
| 378 | RPL7A |  |  | 8592 | -0.395 | -0.3287 | Yes |
| 379 | RPL9 |  |  | 8595 | -0.398 | -0.3236 | Yes |
| 380 | RPS3 |  |  | 8598 | -0.400 | -0.3185 | Yes |
| 381 | RPL8 |  |  | 8602 | -0.405 | -0.3134 | Yes |
| 382 | LSM4 |  |  | 8611 | -0.413 | -0.3088 | Yes |
| 383 | GAPDH |  |  | 8615 | -0.418 | -0.3035 | Yes |
| 384 | RPS27A |  |  | 8620 | -0.422 | -0.2983 | Yes |
| 385 | PPIA |  |  | 8626 | -0.432 | -0.2931 | Yes |
| 386 | RPL39 |  |  | 8640 | -0.443 | -0.2887 | Yes |
| 387 | HMGN2 |  |  | 8644 | -0.449 | -0.2830 | Yes |
| 388 | EEF1A1 |  |  | 8646 | -0.450 | -0.2770 | Yes |
| 389 | RPS19 |  |  | 8648 | -0.453 | -0.2711 | Yes |
| 390 | RPL23A |  |  | 8649 | -0.454 | -0.2649 | Yes |
| 391 | RPS18 |  |  | 8658 | -0.465 | -0.2596 | Yes |
| 392 | RPS25 |  |  | 8661 | -0.467 | -0.2536 | Yes |
| 393 | POLR2L |  |  | 8664 | -0.468 | -0.2475 | Yes |
| 394 | RPL19 |  |  | 8665 | -0.470 | -0.2412 | Yes |
| 395 | RPL37 |  |  | 8667 | -0.473 | -0.2349 | Yes |
| 396 | PHLDA1 |  |  | 8670 | -0.476 | -0.2288 | Yes |
| 397 | RPS28 |  |  | 8676 | -0.491 | -0.2228 | Yes |
| 398 | RPS26 |  |  | 8677 | -0.491 | -0.2161 | Yes |
| 399 | RPS3A |  |  | 8682 | -0.495 | -0.2099 | Yes |
| 400 | RPL13 |  |  | 8685 | -0.499 | -0.2035 | Yes |
| 401 | H3F3B |  |  | 8687 | -0.504 | -0.1968 | Yes |
| 402 | RPL6 |  |  | 8688 | -0.506 | -0.1900 | Yes |
| 403 | RPS23 |  |  | 8690 | -0.506 | -0.1833 | Yes |
| 404 | POLR2J |  |  | 8695 | -0.513 | -0.1769 | Yes |
| 405 | RPLP2 |  |  | 8697 | -0.515 | -0.1701 | Yes |
| 406 | RPS14 |  |  | 8700 | -0.518 | -0.1633 | Yes |
| 407 | RPL30 |  |  | 8701 | -0.522 | -0.1563 | Yes |
| 408 | RPLP0 |  |  | 8703 | -0.524 | -0.1494 | Yes |
| 409 | RPL18A |  |  | 8707 | -0.532 | -0.1425 | Yes |
| 410 | RPL29 |  |  | 8708 | -0.534 | -0.1354 | Yes |
| 411 | TPI1 |  |  | 8709 | -0.538 | -0.1281 | Yes |
| 412 | RPL35 |  |  | 8710 | -0.540 | -0.1208 | Yes |
| 413 | HINT1 |  |  | 8712 | -0.547 | -0.1136 | Yes |
| 414 | RPL32 |  |  | 8715 | -0.556 | -0.1063 | Yes |
| 415 | RPS6 |  |  | 8718 | -0.559 | -0.0990 | Yes |
| 416 | RPS15 |  |  | 8723 | -0.582 | -0.0917 | Yes |
| 417 | SERF2 |  |  | 8724 | -0.594 | -0.0837 | Yes |
| 418 | RPLP1 |  |  | 8728 | -0.606 | -0.0759 | Yes |
| 419 | RPS8 |  |  | 8730 | -0.621 | -0.0676 | Yes |
| 420 | TPT1 |  |  | 8735 | -0.644 | -0.0594 | Yes |
| 421 | RPS2 |  |  | 8739 | -0.668 | -0.0508 | Yes |
| 422 | RPL12 |  |  | 8740 | -0.668 | -0.0418 | Yes |
| 423 | GPX4 |  |  | 8742 | -0.690 | -0.0326 | Yes |
| 424 | RPL41 |  |  | 8743 | -0.718 | -0.0230 | Yes |
| 425 | RPS12 |  |  | 8747 | -0.792 | -0.0126 | Yes |
| 426 | FTH1 |  |  | 8750 | -0.965 | 0.0001 | Yes |
Table: GSEA details [plain text format]

  

Fig 2: RICHARDS\_NOWAKOWSKI\_SCIENCE\_2017\_RG\_EARLY\_UPREG: Random ES distribution      
 Gene set null distribution of ES for **RICHARDS\_NOWAKOWSKI\_SCIENCE\_2017\_RG\_EARLY\_UPREG**
